# Engineering Plant-Derived Exosome-like Nanoparticles as Bioinspired Nanocarriers: From Physicochemical Properties to Tumor Delivery Performance

**DOI:** 10.3390/biomedicines14081689

**Published:** 2026-07-28

**Authors:** Mengru Cai, Yu Qiu, Mingkai Yao, Jiahui Kong, Xiang Li, Qian Zhang, Yiman Jia, Zicheng Zhu, Yukun Zhao, Dong Bai, Yuqin Yang

**Affiliations:** 1 Institute of Basic Theory for Chinese Medicine, China Academy of Chinese Medical Sciences, Beijing 100700, China; caimr@ibtcm.ac.cn (M.C.); yaomingkai2002@163.com (M.Y.); lx15277912206@163.com (X.L.); zhangqian_thu@163.com (Q.Z.); zzczzc0320@163.com (Z.Z.); 2 School of Chinese Materia Medica, Beijing University of Chinese Medicine, Beijing 102488, China; 13666245117@163.com (Y.Q.); kjhtcm@163.com (J.K.); 17692373556@163.com (Y.J.)

**Keywords:** plant-derived exosome-like nanoparticles, natural nanocarriers, drug delivery, cancer therapy, tumor-targeting, nanomedicine, translational challenges

## Abstract

Plant-derived exosome-like nanoparticles (PELNs) are lipid bilayer nanostructures containing endogenous lipids, proteins, nucleic acids, and phytochemicals, which have attracted increasing interest as bioinspired carriers for cancer therapy. This review evaluates how plant source, isolation, purification procedures, vesicle composition, cargo-loading strategy, and administration route shape the quality and tumor-delivery performance of PELNs. The available evidence indicates that plant source and processing are major determinants of particle size, purity, surface charge, cargo profile, and biological activity. Ultracentrifugation remains widely used but is limited by contaminant co-isolation and poor scalability, whereas density-gradient purification and size-exclusion chromatography improve purity, and ultrafiltration and tangential flow filtration offer greater potential for large-scale manufacturing. Passive incubation generally preserves vesicle integrity and is most suitable for hydrophobic small molecules, whereas electroporation, sonication, and extrusion can increase cargo loading but may cause aggregation, membrane remodeling, or loss of endogenous components. Preclinical studies suggest that PELNs can exert intrinsic antitumor effects, modulate the tumor microenvironment, improve chemotherapeutic delivery, and help overcome drug resistance. However, evidence for in vivo tumor-targeting remains less robust than evidence for cellular uptake, and direct comparisons with established nanocarriers remain scarce. Clinical translation will require standardized nomenclature and characterization, reproducible manufacturing, quantitative loading and release assays, route-specific biodistribution studies, and repeated-dose safety evaluation. These findings provide a framework for the rational development of PELNs as reproducible tumor-oriented nanocarriers.

## 1. Introduction

Cancer remains one of the greatest global health burdens [1]. The primary treatment methods include chemotherapy, radiotherapy, surgical resection, and immunotherapy [2]. However, tumor heterogeneity often limits the efficacy of these therapeutic strategies. Furthermore, conventional treatments are frequently accompanied by serious adverse reactions, such as post-surgical immunosuppression and organ toxicity caused by radiotherapy and chemotherapy [3]. Accordingly, there is an urgent need for therapeutic strategies that selectively target tumor cells while minimizing damage to healthy tissues, thereby improving antitumor efficacy, reducing treatment-related toxicity, and prolonging survival. Plant-derived exosome-like nanoparticles (PELNs), derived from plant cells, share structural similarities with mammalian-derived exosomes. They have garnered interest due to their sustainable sourcing and potentially lower immunogenicity compared to certain synthetic or mammalian alternatives [4]. PELNs are generally described as nanoscale vesicle-like particles enclosed by lipid bilayers, with reported diameters commonly ranging from tens to several hundred nanometers. Although they share certain morphological and compositional similarities with mammalian extracellular vesicles, their biogenesis, nomenclature, and subcellular origin in plants remain incompletely defined and may vary among plant species and isolation procedures [5]. PELNs have consequently attracted increasing attention as potential platforms for drug formulation, delivery, and disease treatment.

Over the past few decades, most research on extracellular vesicles (EVs) has focused on those derived from mammalian cells. While this research has yielded significant insights, the clinical translation of mammalian EVs is often hindered by challenges such as potential immunogenicity, the risk of transmitting endogenous pathogenic molecules, and ethical or scalable manufacturing constraints [6]. Moreover, the clinical approval and large-scale production of mammalian cell-derived exosomes may be constrained by source-related, ethical, and manufacturing considerations [7,8]. PELNs exhibit potential advantages over synthetic lipid nanoparticles and liposomes in several key aspects. PELNs offer a dual modality: they provide a structural lipid bilayer delivery platform while naturally co-encapsulating endogenous botanical metabolites [9]. Consequently, PELNs may offer more sustainable production and exert intrinsic pharmacological effects that complement those of the loaded therapeutic cargo. Furthermore, owing to their diverse botanical origins, certain PELNs have demonstrated efficient cellular uptake and favorable targeting potential in preliminary models. PELNs also enhance drug delivery when loaded with chemotherapeutics, nucleic acids, or immunomodulators, improving precision and reducing off-target effects [10]. Similarly, certain ginseng-derived nanovesicles have been reported to cross the blood–brain barrier in specific murine models [11]. Furthermore, plant sources offer a scalable and cost-effective foundation for EV production.

The nomenclature used for PELNs remains inconsistent because their biogenesis and subcellular origins have not been fully established. Terms such as plant extracellular vesicles, plant-derived nanovesicles, and plant-derived exosome-like nanoparticles have therefore been used interchangeably in the literature. For consistency, this review uses the operational term PELNs to describe the plant-derived vesicle-like nanoparticles discussed throughout the manuscript. The term “plant extracellular vesicles” (PEVs) is retained only when referring to studies that specifically used this designation or directly isolated extracellular vesicles from extracellular fluids. In this review, “engineered PELNs” refers specifically to PELNs modified through exogenous cargo loading, surface functionalization, membrane hybridization, or related formulation strategies.

Although PELNs have been increasingly investigated as natural nanocarriers, several issues still limit their development as reproducible tumor drug delivery systems. First, many studies describe PELNs according to plant source or disease model, but fewer analyses connect the plant matrix, isolation workflow, vesicle composition, and final delivery performance. Second, the effects of preparation-dependent quality attributes, including size distribution, surface charge, purity, lipid composition, protein profile, and residual plant-derived impurities, remain insufficiently discussed from a nanomaterial engineering perspective. Third, most loading and targeting strategies are evaluated case by case, whereas general design principles for selecting loading methods, administration routes, and tumor models are still lacking. Recent studies and reviews have summarized the biological features, isolation strategies, therapeutic potential, and drug-delivery applications of exosomes, plant-derived exosome-like nanoparticles, and related herbal or dietary nanovesicles [12,13,14,15,16,17,18].

**Figure 1:**
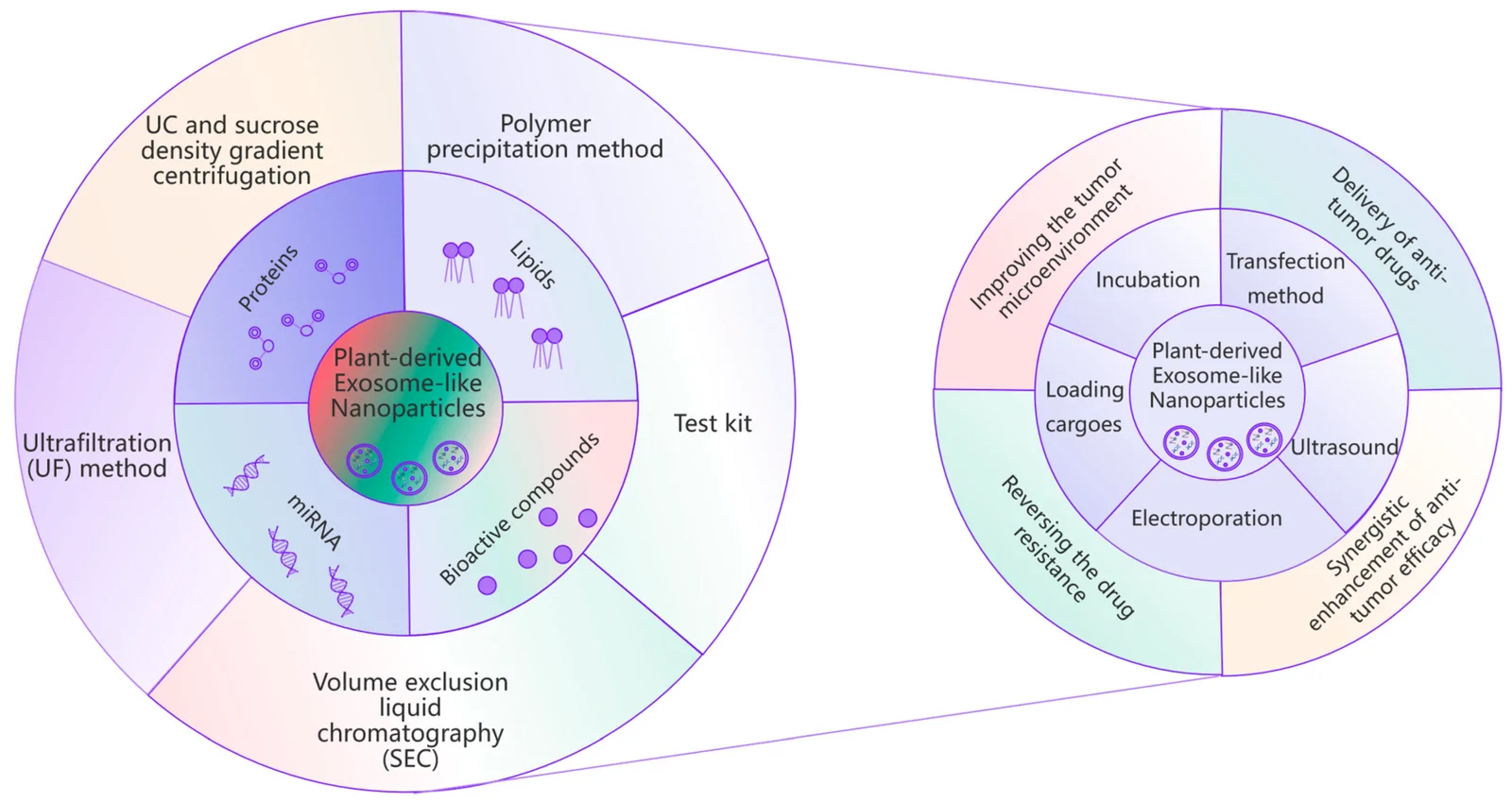
The drug delivery and therapeutic application of plant-derived exosome-like nanoparticles in tumor treatment.

Therefore, this review discusses PELNs as bioinspired nanocarriers for tumor drug delivery from a source-processing-structure-performance perspective. We first summarize how plant source and isolation strategy influence vesicle quality attributes. We then analyze the roles of lipids, proteins, nucleic acids, and phytochemicals in vesicle stability, cellular uptake, intrinsic bioactivity, and cargo delivery. Subsequently, we compare representative drug-loading approaches and discuss antitumor applications in tumor microenvironment regulation, chemotherapeutic delivery, drug resistance reversal, and combination therapy. Finally, we highlight the translational barriers that must be addressed before PELNs-based formulations can move toward standardized manufacturing and clinical evaluation. The overall design logic of PELNs as tumor-oriented nanocarriers is summarized in Figure 1, including plant source selection, vesicle isolation, intrinsic cargo composition, drug-loading strategies, tumor-targeting behavior, and therapeutic applications.

### Literature Search Strategy

This article was prepared as a narrative review. Relevant studies were collected from PubMed, Web of Science, Scopus, and Google Scholar using combinations of the following terms: “plant-derived exosome-like nanoparticles”, “plant extracellular vesicles”, “plant-derived nanovesicles”, “drug delivery”, “tumor”, “cancer therapy”, “cargo loading”, “targeting”, “oral delivery”, “intravenous delivery”, and “nanomedicine”. Priority was given to original research articles and recent reviews that reported isolation methods, physicochemical characterization, cargo composition, loading strategies, antitumor mechanisms, biodistribution, safety assessment, or translational challenges. Studies unrelated to plant-derived vesicle-like nanoparticles or lacking sufficient characterization information were not discussed in detail.

## 2. Extraction and Isolation Methods for PELNs

PELNs can be isolated from different plant tissues, fruits, herbs, and edible plant-derived materials, but their physicochemical properties and biological activities are strongly influenced by the selected plant source and isolation workflow. Commonly used methods include differential ultracentrifugation, density-gradient ultracentrifugation, polymer precipitation, size-exclusion chromatography (SEC), ultrafiltration or tangential flow filtration (UF/TFF), commercial kit-based isolation, and emerging microfluidic or affinity-based approaches [12,19]. From a nanomaterial engineering perspective, isolation is not merely a preparative step; it determines critical quality attributes such as particle size distribution, vesicle integrity, surface charge, purity, residual soluble proteins, co-isolated polysaccharides, pigment contamination, and batch-to-batch reproducibility. PELNs preparation generally involves plant tissue disruption or infiltration, preliminary clarification, vesicle enrichment, and further purification.

Therefore, method selection should be guided by the intended downstream application. Mechanistic and omics studies require high purity and minimal contamination, whereas drug delivery studies require preserved vesicle integrity, colloidal stability, scalable recovery, and compatibility with cargo loading and safety assessment. No single method is optimal for all PELNs preparations, and combined workflows are often needed to balance yield, purity, structural preservation, and scalability [13,14,15,16,17,18].

### 2.1. Ultracentrifugation and Sucrose Density-Gradient Centrifugation

Historically regarded as a standard method for EV isolation, ultracentrifugation (UC) remains widely used in PELNs studies because it does not require precipitation reagents and can process crude plant extracts after preliminary clarification [15]. However, its practical throughput is constrained by rotor capacity, long processing time, high equipment cost, and operator-dependent variability. Repeated high-speed centrifugation may also lead to vesicle loss, aggregation, deformation, or co-pelleting of protein aggregates, polysaccharides, and other non-vesicular components. These issues are particularly relevant for plant-derived samples, in which soluble proteins, pigments, starch, and cell wall-derived materials may sediment together with vesicle-like particles [20,21,22,23,24,25]. Therefore, UC is useful for laboratory-scale enrichment and comparative mechanistic studies, but it is usually insufficient as a stand-alone strategy for translational PELNs production. For drug delivery applications, UC is preferably combined with density-gradient purification, SEC, or UF/TFF to improve purity, reduce soluble contaminants, and preserve vesicle-associated bioactivity [23,24,25].

### 2.2. Polymer Precipitation Method

Hydrophilic polymers such as polyethylene glycol (PEG) reduce the solubility of vesicle-like particles and promote their precipitation from solution. This method is simple, inexpensive, and does not require specialized equipment [26]. However, it commonly co-precipitates non-vesicular contaminants and produces dilute preparations that may require an additional concentration step. Emmanuela et al. successfully isolated spherical PELNs from *Solanum nigrum* using differential centrifugation followed by PEG precipitation; the particles had an average diameter of 107 nm [27]. Yoon et al. isolated uniformly spherical green onion-derived exosome-like nanoparticles (GDENs) using a modified PEG precipitation method, achieving a high particle yield while maintaining preparation quality [28]. Zhang et al. optimized PEG concentration, pH, and centrifugation temperature to maximize the yield and preserve the biological activity of Tartary buckwheat-derived nanoparticles (TBDNs) [29]. Kalarikkal et al. developed a method to purify ginger nanovesicles by polymer precipitation (using PEG 6000). This method can significantly reduce the cost of nanovesicle purification compared with UC and may be applicable to nanovesicles derived from various dietary plants [30]. Higher-purity PELNs preparations can be obtained by further purifying the precipitate through sucrose density-gradient centrifugation. Iriawati et al. optimized the separation process of papaya PELNs by differential centrifugation and PEG methods [31]. Vanessa et al. successfully isolated spherical goldenberry-derived exosome-like nanoparticles with an average diameter of 227.7 nm using differential centrifugation combined with precipitation in 12% PEG 6000 [32]. Kusnandar et al. combined PEG-based precipitation, sequential filtration, and a series of low-temperature centrifugation steps to isolate spherical PELNs from yam bean roots [33]. Polymer precipitation is simple, inexpensive, and suitable for rapid enrichment of vesicle-like nanoparticles from plant extracts. However, PEG-based precipitation often co-isolates soluble proteins, polysaccharides, nucleic acids, pigments, and other non-vesicular materials, which may interfere with downstream functional assays and cargo analysis [27,28,29,30,31,32,33]. Therefore, this method is more appropriate for preliminary screening or high-yield enrichment than for therapeutic-grade PELNs preparation. When higher purity is required, PEG precipitation should be followed by polishing steps such as SEC, density-gradient ultracentrifugation, or membrane-based purification [13,30,31,32,33].

### 2.3. Size-Exclusion Chromatography

Size-exclusion chromatography (SEC) separates vesicles according to their hydrodynamic size using a porous stationary phase. Larger vesicle-like particles elute earlier because they are excluded from the pores, whereas smaller soluble proteins and metabolites enter the pores and elute later [34]. Compared with precipitation-based methods, SEC better preserves vesicle integrity and biological activity and is effective for removing soluble contaminants. However, SEC usually requires sample pre-concentration, may reduce total vesicle recovery, and has limited loading capacity per column. Thus, SEC is particularly useful as a polishing step after UC, UF/TFF, or precipitation-based enrichment [35,36,37,38,39,40,41].

**Figure 2:**
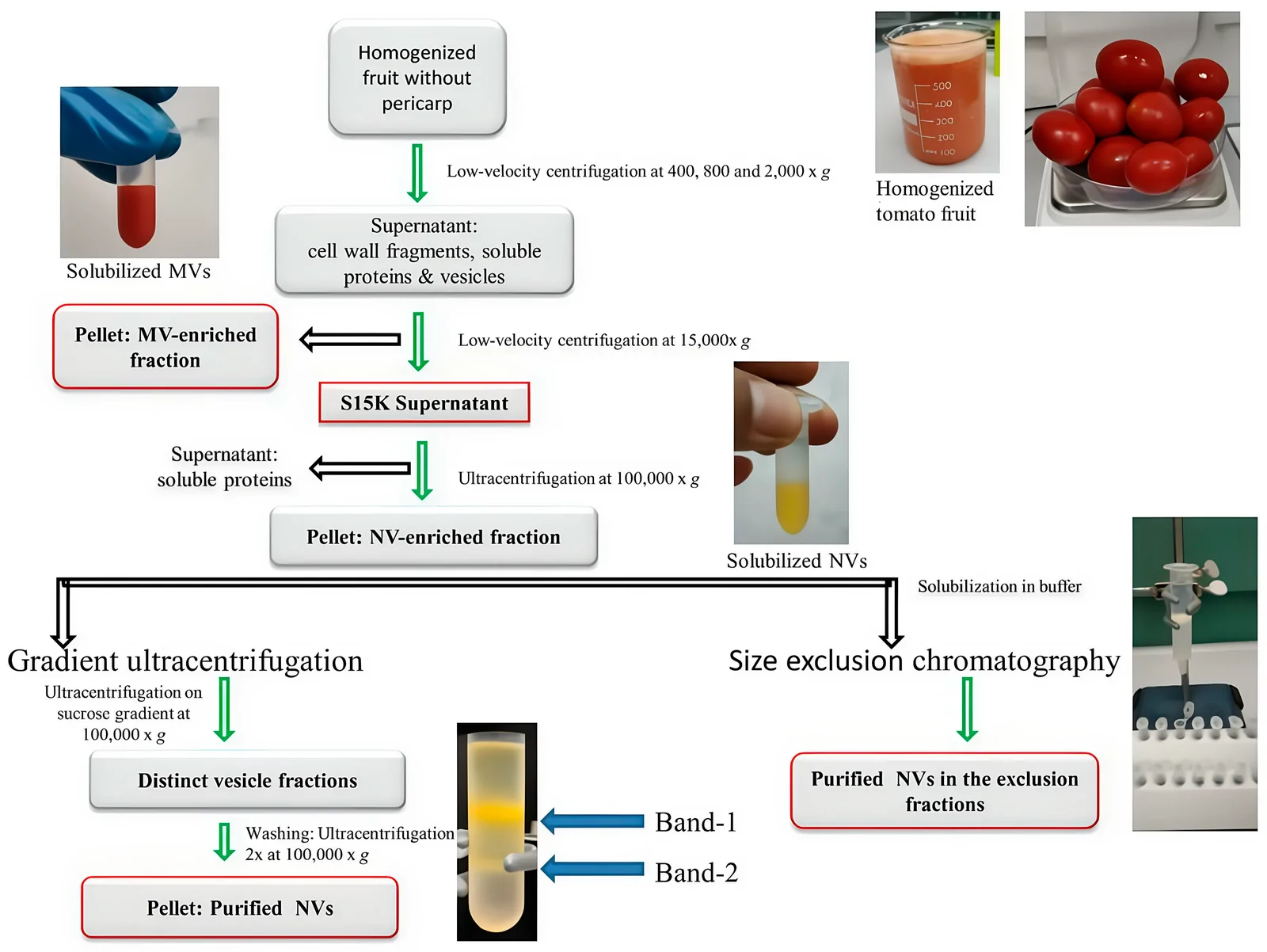
Isolation and purification workflow for tomato fruit-derived microvesicles (MVs) and nanovesicles (NVs). Differential ultracentrifugation was used for vesicle enrichment, followed by gradient ultracentrifugation or size-exclusion chromatography for further purification. Reproduced from Bokka et al. [38] under the Creative Commons Attribution 4.0 International License (CC BY 4.0).

You et al. introduced SEC combined with ultrafiltration for the isolation of PELNs from cabbage [35]. SEC has also been used to isolate carrot-derived PELNs [36]. Kantarcıoğlu et al. isolated coffee-derived PELNs by SEC using a column packed with Sepharose CL-6B and phosphate-buffered saline (PBS) as the eluent [26]. Giritlioglu et al. isolated PELNs with oval-to-elliptical morphologies from Viburnum opulus using a combination of centrifugation, filtration, and chromatographic column methods [37]. Bokka et al. used differential ultracentrifugation (DUC) for the large-scale enrichment of tomato-derived PELNs, followed by either sucrose density-gradient ultracentrifugation (GUC) or SEC for further purification. In comparing the performance of the two methods, GUC proved to be superior to SEC in separating different vesicle populations based on buoyancy density (in contrast to SEC, which is better at removing co-purified proteins and other impurities). It is essential to note that both methods require concentration of the sample to carry out the separation [38]. Leng et al. collected and purified blueberry PELNs by SEC [39]. Sánchez-López et al. used SEC to isolate pomegranate PELNs. This method yielded highly enriched PELNs with fewer co-isolated non-vesicular proteins [40]. Vestuto et al. used DUC and SEC to isolate PELNs from conditioned media of hairy-root (HR) cultures of the medicinal plant *Salvia* miltiorrhiza [41]. Although time-consuming, this workflow is broadly applicable and technically straightforward. Its principal advantage is the recovery of relatively homogeneous and structurally intact PELNs with high purity, although the overall yield is generally low. The isolation and purification workflow for tomato-derived PELNs, combining differential ultracentrifugation with sucrose density-gradient ultracentrifugation or size-exclusion chromatography, is illustrated in Figure 2 [38].

### 2.4. Commercial Kit-Based Isolation

Commercial kit-based isolation provides simplified workflows for enriching vesicle-like nanoparticles, but the underlying separation principles vary among products and may include polymer precipitation, affinity capture, membrane-based enrichment, or proprietary formulations. These kits are convenient for small-scale exploratory studies because they require less specialized equipment and are easy to operate. However, their limited methodological transparency, high cost, variable purity, and poor scalability make it difficult to compare results across studies or to establish standardized manufacturing workflows. In addition, kit-derived preparations may contain co-isolated nucleic acids, proteins, or reagent residues, which should be carefully evaluated before functional interpretation [26,42,43,44].

### 2.5. Ultrafiltration Method

Ultrafiltration (UF) and tangential flow filtration (TFF) separate particles according to membrane molecular weight cut-off or pore size. These approaches are attractive for PELNs preparation because they are relatively rapid, reagent-free, scalable, and compatible with concentration or buffer exchange. Compared with dead-end filtration, TFF can reduce membrane clogging and improve processing of larger sample volumes. However, membrane adsorption, concentration polarization, shear stress, and pore-size-dependent loss may affect vesicle recovery, morphology, and functional integrity. Therefore, filtration parameters such as membrane type, cut-off value, pressure, flow rate, and processing time should be reported and optimized for each plant source [45,46,47,48].

Beyond conventional techniques, charge-based methods such as electrophoresis have been explored for selected PELNs preparations [49,50]. Electrophoresis exploits the negative surface charge of lipid vesicles to separate them from neutral or positively charged contaminants without applying high shear forces. Although this approach can be rapid and may preserve vesicle integrity, its low throughput limits large-scale manufacturing. Microfluidic technologies can separate PELNs according to their size, charge, deformability, or affinity characteristics but are currently more suitable for analytical and diagnostic applications than for large-scale production. Immunoaffinity capture uses antibodies immobilized on magnetic beads to bind selected vesicle-associated surface proteins. This method offers high specificity and purity but remains expensive and is limited by the lack of validated universal plant vesicle markers. In addition, incomplete removal of affinity reagents may affect vesicle integrity and downstream applications [12]. Lo et al. studied the effects of different isolation methods on the traits of lotus leaves, using the leaves as a source of PELNs, and evaluated their potential bioactivities [51]. Given the advantages and limitations of individual isolation methods, combined workflows may provide a more appropriate balance between yield and purity. Jang et al., for example, evaluated a workflow combining ultracentrifugation with ExoQuick-based precipitation [52]. To overcome these challenges, Suresh, A. P. et al. have recently developed a polyethylene glycol (PEG) 6000-based method for cost-effective purification of ginger PELNs, with comparable efficiency to differential ultracentrifugation [53]. Steć, et al. developed a workflow combining differential centrifugation, filtration, and size-exclusion chromatography for the isolation of PELNs from mango pulp [54].

Differential ultracentrifugation remains the most widely applied approach in plant systems due to its ability to process large tissue volumes; however, it frequently co-isolates plant proteins, polysaccharides, and secondary metabolites. In contrast, SEC and Density-gradient ultracentrifugation(DG-UC) provide improved purity but at the expense of reduced throughput and vesicle recovery. Immunoaffinity and microfluidic-based methods offer high specificity and analytical precision, yet their limited scalability and lack of validated universal plant EV markers restrict their broader application. Therefore, method selection should be guided by downstream application requirements rather than assumed methodological superiority [55]. Unlike mammalian cells, plant tissues contain cell wall components (cellulose, pectin, hemicellulose) [56]. and abundant interfering metabolites (starch, pigments, polyphenols, secondary metabolites) that can significantly affect PELNS isolation [57]. Cellulose and pectin form rigid structures that hinder vesicle release, while starch granules and pigments can co-precipitate with PELNs, reducing purity and yield and potentially impairing vesicle function [58]. Overall, the isolation strategy should be selected according to the intended application rather than methodological convenience alone. UC and PEG precipitation are useful for enrichment, SEC and density-gradient purification are suitable for improving purity, and UF/TFF is more compatible with scalable production. For mechanistic studies, high purity and compatibility with proteomic, lipidomic, and RNA analyses are essential. For drug delivery studies, vesicle integrity, colloidal stability, sterility, cargo-loading compatibility, and reproducibility should be prioritized. Future PELNs studies should report not only size and zeta potential, but also yield, protein-to-particle ratio, residual contaminants, storage conditions, and functional retention after isolation [49,50,51,52,53,54,55,56,57,58]. Table 1 lists the extraction methods and characteristics of different PELNs.

## 3. Composition

PELNs are mainly composed of biomolecules such as lipids, proteins, nucleic acids, and bioactive compounds [4]. Each class of PELNS constituents performs a distinct biological function. The protein profile determines, to a certain extent, the mechanism by which PELNs are taken up by cells [66], the lipid component plays a key role in achieving efficient cellular uptake of PELNs [67], and miRNAs (microRNAs) regulate the expression of genes in the cells where PELNs are taken up [68]. Despite increasing interest in the protein composition of PELNs, universally accepted plant-specific EV markers have not yet been established, and comprehensive reference databases remain incomplete for many plant species. Large-scale comparative proteomic analyses across different plant sources are needed to identify conserved vesicle-associated proteins, distinguish genuine PELNs components from co-isolated contaminants, and provide a more reliable basis for PELNs classification, quality control, and functional interpretation [4,66,67,68].

### 3.1. Proteins

Proteins are important functional components of PELNs and may influence vesicle stability, cellular uptake, intercellular communication, and biological activity. Reported PELNs-associated proteins include cytoplasmic proteins, stress-response proteins, enzymes, ribosomal proteins, membrane proteins, transporters, and channel-related proteins. From an engineering perspective, these proteins are relevant for two reasons: they may contribute to intrinsic bioactivity or tissue interaction, and they may serve as potential quality-control markers. However, soluble plant proteins can also co-isolate with vesicles, making it necessary to distinguish vesicle-associated proteins from non-vesicular contaminants [69,70,71,72,73,74]. Overall, proteomic analysis can support PELNs identification, functional annotation, and quality evaluation, but current datasets remain fragmented because plant species differ substantially in genome annotation quality and protein database completeness. Future studies should combine proteomic profiling with vesicle purification controls, enzymatic protection assays, and functional validation to determine whether identified proteins are genuinely vesicle-associated and whether they contribute to tumor-targeting, immune modulation, or cellular uptake [70,71,72,73,74]. However, these overlapping proteins should be interpreted cautiously because differences in plant species, purification methods, and proteomic workflows may affect protein detection and annotation.

### 3.2. Lipids

The lipid bilayer structure and lipid content of PELNs are crucial for their transport capacity, stability of their contents, and promotion of uptake. Most PELNs membranes are enriched in phosphatidic acid (PA), phosphatidylcholine (PC), dilactosyl diacylglycerol (DGDG), phosphatidylethanolamine (PE), and mono-galactosyl diacylglycerol (MGDG). Unlike mammalian exosomes, which are often enriched in cholesterol and sphingolipids, PELNs tend to contain higher proportions of plant-specific phospholipids and glycolipids. Nevertheless, PELNs from different sources exhibit variation in lipid composition. The lipid-rich structure of PELNs may support drug delivery by improving cargo solubility, enabling membrane-associated encapsulation, enhancing bioavailability, and reducing off-target toxicity. Chen et al. concluded that PC, triglycerides (TG), and PE were the major lipid components of TFENs from Camellia sinensis [60]. Tangerine-derived nanovesicles (TNVs) were enriched in lipids, citrullinated compounds, and flavonoid derivatives [72]. Poria-derived PELNs (PCELNs) were mainly composed of TG, ceramides, PE, diglycerides, and di-lactosyl monoacylglycerol [75]. Edible kiwifruit-derived extracellular vesicles (KEVs), which contain sphingoid base phosphates, sphingoid bases, and sphingomyelins, have demonstrated favorable biocompatibility, controllable size, and good stability. Their surfaces can also be functionalized with aptamers for targeted small interfering RNA (siRNA) delivery [76].

Despite the significant value of the lipid components of PELNs in both biological research and practical applications, our understanding of their specific components and the mechanisms underlying their functions remains limited. Consequently, there is a pressing need for more comprehensive and systematic investigations to fully elucidate the potential value of exosomal lipids in plants. It is important to highlight that not all PELNs contain cholesterol. Several studies have shown that the lipid components of PELNs can exert notable bioactive effects on recipient cells. Therefore, detailed profiling of each lipid component and a precise elucidation of their mechanisms of action are crucial for the development of effective PELNs-based therapeutic strategies.

Although PELNs are highly enriched in phospholipids and lack prominent cholesterol accumulation characteristic of mammalian exosomes, lipidomic profiling verified that cholesterol is not entirely absent from PELNs but is only present at trace levels in a minor fraction of vesicles [77]. Lipidomic analyses of PELNs from tomato, grape, grapefruit, tea, and other plant sources indicate that purified vesicle-like nanoparticles are lipid-rich, although their lipid profiles vary according to plant species, tissue type, extraction procedure, and purification method [78]. Frequently reported lipid classes include PA, PC, PE, MGDG, and DGDG, but their relative abundance is not universal. Therefore, lipid composition should be treated as a source-dependent quality attribute rather than a fixed signature of all PELNs [60,72,75,76,77,78].

### 3.3. MicroRNAs

MicroRNAs (miRNAs) are small non-coding RNAs of approximately 18–24 nucleotides that regulate gene expression mainly through mRNA cleavage, translational repression, or other post-transcriptional mechanisms. PELNs are also known for their specificity, low immunological risk, and, most importantly, their high bioavailability. In terms of safety, plants do not contain any zoonotic or human pathogens. Higher bioavailability is one of their most interesting features in cross-kingdom interactions: components packaged in PELNs, including miRNAs, are protected from degradation by the lipid layer [79]. Plant miRNAs enclosed in PELNs have been proposed to participate in cross-kingdom regulation by interacting with mammalian transcripts; however, this hypothesis remains controversial. Technical issues related to RNA extraction, sequencing depth, contamination control, dietary dose relevance, target prediction, and functional validation make it difficult to determine whether plant miRNAs exert reproducible regulatory effects in mammalian systems, particularly in cancer models [4,79,80,81,82,83,84].

For RNA-related cargo, the key issue is not only whether small RNAs can be detected in PELNs preparations, but also whether they are protected within vesicles, delivered to recipient cells at biologically relevant levels, and capable of inducing sequence-specific effects. Future studies should include appropriate RNase protection assays, vesicle-disruption controls, spike-in normalization, contamination assessment, and validation of predicted mammalian targets. These controls are essential for distinguishing true PELNs-mediated RNA delivery from technical artifacts or co-isolated free RNA [4,83,84].

### 3.4. Bioactive Compounds

**Figure 3:**
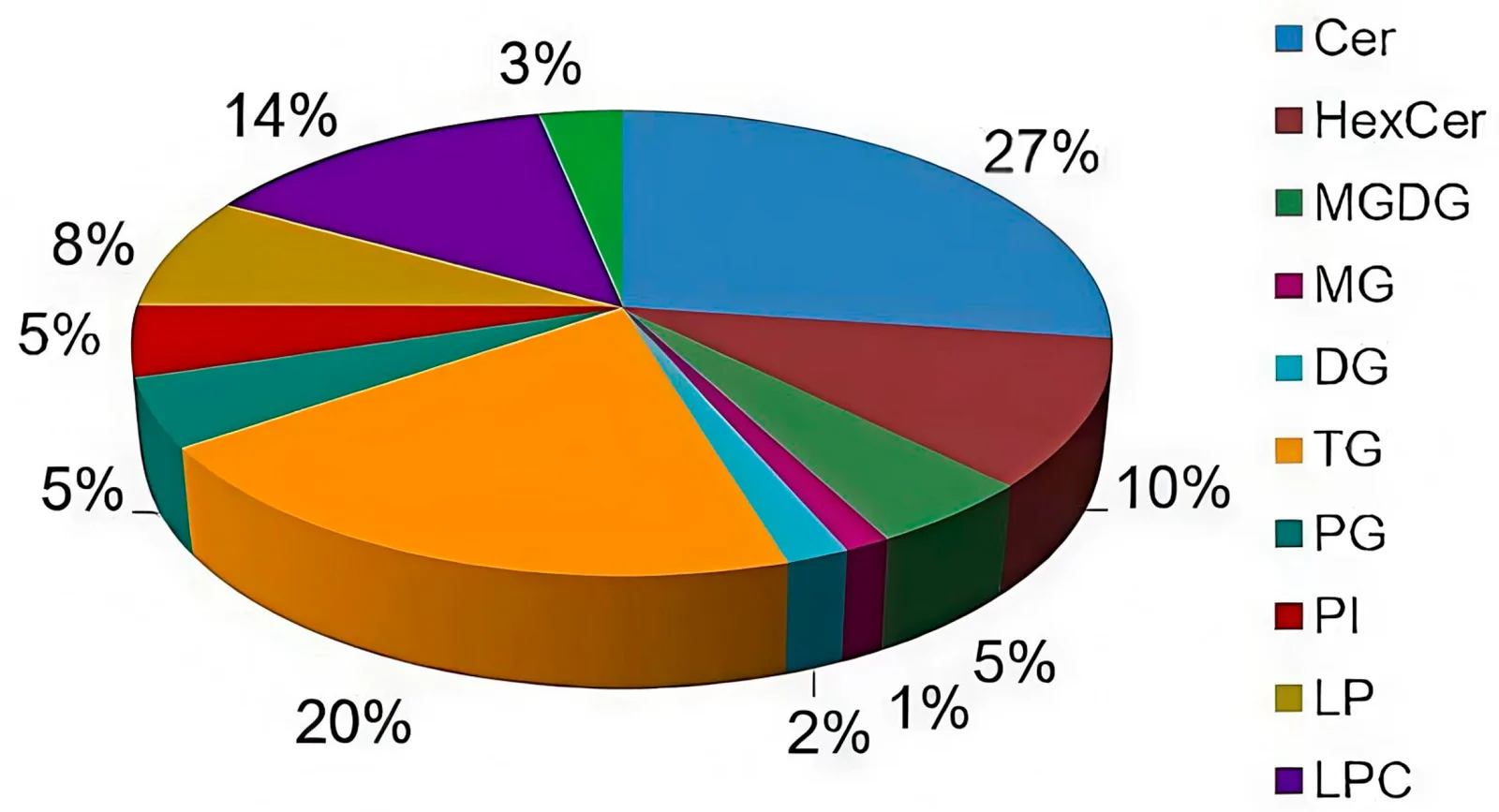
Lipid composition of *Morus nigra* L. leaf-derived lipid nanoparticles (MLNPs). Cer, ceramides; HexCer, hexosylceramides; MGDG, monogalactosyldiacylglycerol; MG, monoglycerides; DG, diglycerides; TG, triglycerides; PG, phosphatidylglycerol; PI, phosphatidylinositol; LP, lysophospholipids; LPC, lysophosphatidylcholine. Reproduced from Gao et al. [89] under the Creative Commons Attribution 4.0 International License (CC BY 4.0).

PELNs contain plant-specific active derivatives due to their unique origin. For example, black beans contain plant-derived secondary metabolites, including saponins and flavonoids, with potential nutritional and pharmacological activities [85]. Edible ginger-derived nanoparticle active ingredients, including 6-gingerol and 6-shogaol, which have multiple molecular targets, including inflammatory mediators, could be used in new therapeutic approaches for the prevention and treatment of inflammatory bowel disease and colon-related cancers [86]. Tajik et al. were the first to isolate PELNs from two *Cannabis sativa* chemotypes and evaluate their anticancer effects [87]. Cucumber-derived nanovesicles (CDNVs) are natural nanocarriers for cucurbitacin B [88]. Mulberry-derived lipid nanoparticles contain various functional components, including lipids, proteins, and flavonoids, and galactose was identified as a potential mediator of liver tumor-targeting [89]. Lipidomic analysis further showed that *Morus nigra* L. leaf-derived lipid nanoparticles (MLNPs) were mainly composed of ceramides (27%), triglycerides (20%), lysophosphatidylcholine (14%), and hexosylceramides (10%) (Figure 3) [89]. *Aloe vera*-derived PELNs may improve cargo bioavailability by protecting encapsulated molecules from degradation within their phospholipid bilayer [90].

Understanding the endogenous bioactive components of PELNs is essential for evaluating their dual role as natural therapeutic entities and drug delivery carriers. Unlike inert synthetic nanocarriers, PELNs may carry plant-derived metabolites, lipids, proteins, and RNAs that contribute to intrinsic biological activity [85,86,87,88,89,90]. However, this feature also complicates mechanistic interpretation because the observed effects may arise from vesicle structure, endogenous cargo, exogenous drug loading, or co-isolated plant components. Therefore, future studies should combine compositional profiling with depletion, fractionation, and functional validation experiments to clarify which components are responsible for specific antitumor or immunomodulatory effects. Representative PELNs compositions and biomedical activities are summarized in Table 2 [18].

## 4. Drug-Loading Strategies and Surface Functionalization of PELNs

**Figure 4:**
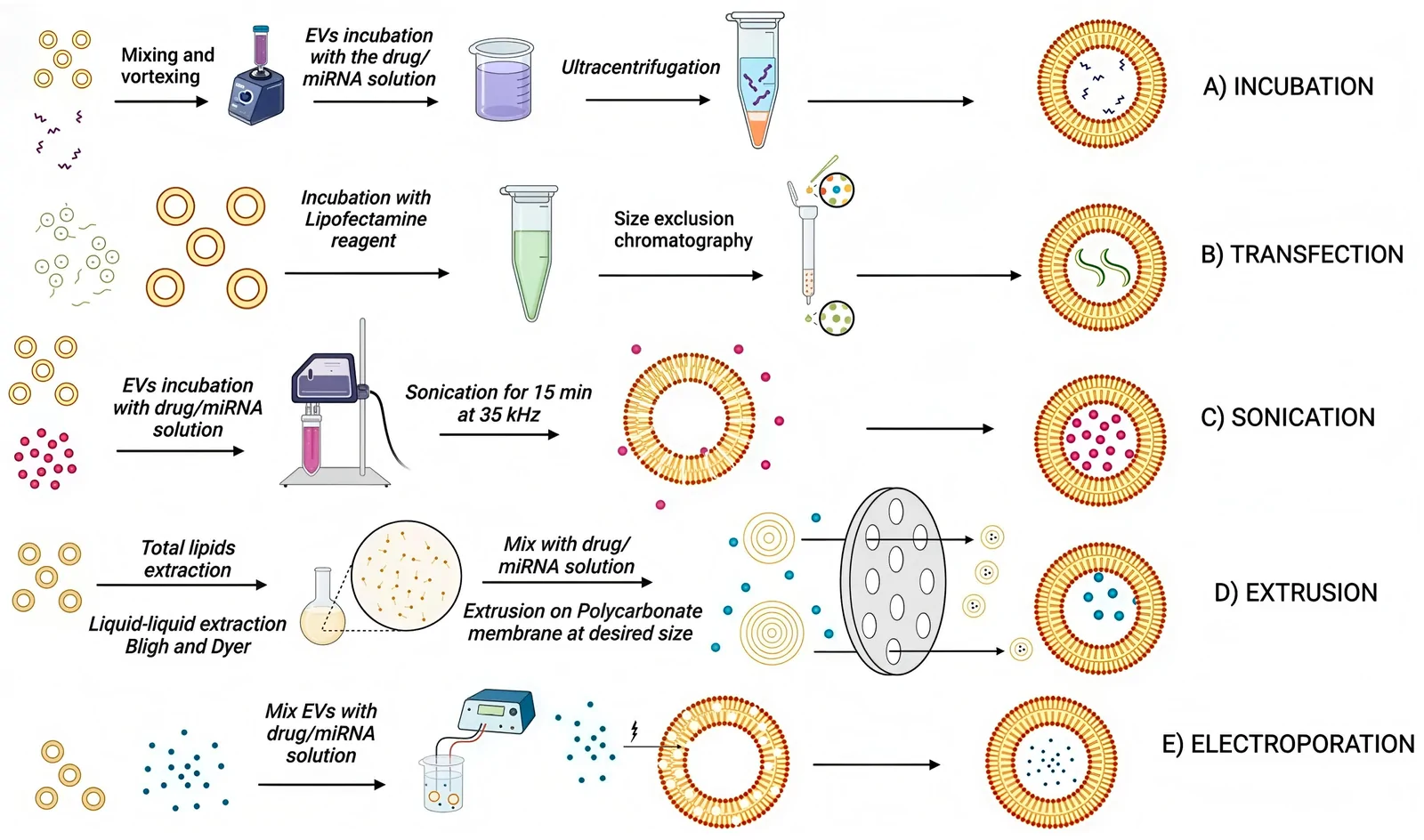
Strategies for exosome drug loading. Reproduced from Langellotto et al. [58] under the Creative Commons Attribution 4.0 International License (CC BY 4.0). The symbols and colors are used for schematic and visual differentiation only and do not represent quantitative information.

Efficient cargo loading is central to the development of PELNs as drug delivery systems. In general, loading strategies can be divided into passive loading and active loading. Passive loading mainly relies on co-incubation between PELNs and therapeutic molecules, whereas active loading transiently disrupts or remodels the vesicle membrane through electroporation, sonication, extrusion, freeze–thaw cycles, pH adjustment, or related approaches [94,95]. Each strategy affects loading efficiency, vesicle integrity, colloidal stability, release kinetics, and biological activity differently. Therefore, loading efficiency should not be evaluated as an isolated parameter; it should be interpreted together with particle size, zeta potential, morphology, aggregation state, cargo leakage, retained bioactivity, and batch reproducibility [94,95]. Table 3 lists the latest research on nanoparticles derived from grapefruit, ginger, lemon, grape, and broccoli, including engineering strategies, loading efficiency, targeting methods, and therapeutic effects. Representative cargo-loading strategies are summarized in Figure 4, including incubation, transfection-related loading, sonication, extrusion, and electroporation. These approaches differ in membrane perturbation intensity, cargo compatibility, loading efficiency, and their potential effects on vesicle integrity.

### 4.1. Incubation for Drug Loading

Co-incubation is the simplest passive loading strategy, in which isolated PELNs are mixed with small molecules or therapeutic agents for a defined period. This approach is particularly suitable for hydrophobic or amphiphilic molecules that can partition into the lipid bilayer or vesicle-associated lipid domains. Its main advantage is that it avoids strong physical disruption and therefore better preserves vesicle morphology and membrane integrity [95,96]. However, loading efficiency is often limited and depends on cargo hydrophobicity, molecular size, concentration gradient, incubation time, temperature, and vesicle composition. Similar passive loading principles have also been investigated in other nanoparticle systems, including mesoporous silica nanoparticles and albumin-based nanoparticles, which provide methodological references for small-molecule encapsulation but should not be directly interpreted as PELNs-specific evidence [96,97]. PELNs have been explored for the loading of representative small molecules such as curcumin and other poorly water-soluble compounds [98,99,100,101,102]. For PELNs-based delivery, co-incubation should be accompanied by removal of free drug and quantitative assessment of encapsulation efficiency, loading capacity, release behavior, and vesicle stability [98,99,100,101,102].

### 4.2. Electroporation for the Delivery of Drug Molecules

Electroporation is a simple and effective method for loading drugs into PELNs. It maintains the original properties of the drugs and provides higher loading efficiency than other methods. It is relatively safe as it does not require special chemical reagents. More importantly, this new approach is not limited to specific cells and addresses the challenges of loading large amounts of mRNA. PELNs-targeted delivery of DNA can be used for transgenic therapy and can also be loaded by different methods [103]. In addition, the loading efficiency varies with changing electroporation conditions. The principle of electroporation is to load small-molecule drugs into PELNs by applying an external electric field and using short high-voltage pulses to disrupt the membrane barrier. CCL2-siRNA was loaded into PELNs by electroporation [104]. The modified PELNs still maintained the fundamental properties of PELNs and showed good targeting and therapeutic effects in vitro and in vivo. Ma et al. prepared a new neutrophil membrane-engineered ginseng root PELNs and N-exo-miRNA182-5p. miRNA182-5p was transduced into the N-exo by the electroporation technique to obtain N-exo-miRNA182-5p [105]. Restricted delivery of macromolecules is a limitation. Zhu et al. used an electroporation method to prepare tumor-exocytosed exosome/AIE luminogen (AIEgen) hybrid nanovesicles (DES) that could facilitate effective tumor penetration [106]. Mammadova et al. performed electroporation using two different ratios of tomato PELNs to tolvaptan [107]. Electroporation can improve the loading of nucleic acids and other hydrophilic cargos by generating transient membrane pores under an external electric field [103,104,105,106,107]. However, its application to PELNs should be interpreted cautiously because electroporation may induce vesicle aggregation, membrane destabilization, cargo precipitation, or loss of vesicle-associated components. Optimization of voltage, pulse duration, buffer composition, vesicle concentration, and cargo-to-vesicle ratio are, therefore, essential. In addition, some electroporation examples are derived from mammalian EV systems and should be used only as methodological references unless validated directly in plant-derived vesicles [103,104,105,106,107].

### 4.3. Sonication-Assisted Cargo Loading

Sonication uses acoustic energy and mechanical shear to transiently perturb vesicle membranes, thereby facilitating cargo entry into PELNs. Compared with simple incubation, sonication may improve the loading of poorly permeable compounds, but it can also alter vesicle morphology, promote aggregation, and affect membrane-associated proteins [108,109,110]. Therefore, sonication-loaded PELNs should be characterized before and after loading using particle size analysis, zeta potential measurement, electron microscopy, cargo quantification, and functional assays. This is particularly important when the therapeutic effect depends on intact vesicle structure or endogenous bioactive components [109,110]. Sonication is technically straightforward and relatively inexpensive and may improve cargo-loading efficiency. Nevertheless, because it transiently disrupts the PELNs membrane, excessive acoustic energy may compromise vesicle integrity and endogenous biological activity. Sonication conditions should, therefore, be optimized according to the stability requirements of the intended formulation.

### 4.4. Extrusion for Cargo Loading

In this method, a mixture of the drug and PELNs is repeatedly passed through polycarbonate membrane filters of different sizes (100–400 nm) at controlled temperatures. This process promotes drug binding to PELNs and enables efficient encapsulation of new drug molecules. The strategy can also be extended to membrane hybridization of PELNs. The method is capable of obtaining vesicle-like structures with PELNs-like properties in large quantities. Kim et al. used balloon flower root PELNs (BDEs) to synthesize multilayered liposome-loaded silymarins-hybridized BDEs by simple membrane hydration followed by membrane extrusion [47]. Mammadova et al. encapsulated tolvaptan by extrusion and electroporation [107]. Extrusion can improve contact between cargo molecules and vesicle membranes and may also be used to generate hybrid vesicle-like structures. Its advantages include relatively short processing time, controllable size distribution, and compatibility with membrane hybridization strategies [47,107]. However, repeated extrusion may alter membrane organization, remove or redistribute vesicle-associated proteins, and change the biological identity of native PELNs. Therefore, extrusion-based loading is more suitable when controlled formulation properties are prioritized, whereas native vesicle bioactivity should be carefully re-evaluated after processing [47,107].

### 4.5. Transfection-Related and Source-Cell Engineering Approaches

Transfection-related strategies and source-cell engineering approaches aim to modify vesicle cargo before or after vesicle isolation. In principle, donor cells or source tissues can be engineered to express specific nucleic acids, peptides, or proteins, which may then be incorporated into secreted vesicles. Alternatively, isolated vesicles can be loaded with nucleic acids using liposome-assisted methods or other post-isolation approaches [111,112]. These strategies provide opportunities for functional engineering, especially for RNA delivery, but their application to PELNs remains more complex than that of mammalian EVs because plant donor systems, tissue heterogeneity, and purification workflows are less standardized. For PELNs, transfection-related strategies should be discussed cautiously because stable genetic manipulation of plant donor tissues, post-isolation RNA loading, and liposome-assisted cargo insertion represent different technical routes with distinct biosafety, scalability, and regulatory implications [111,112]. In addition, residual transfection reagents, free nucleic acids, and non-vesicular complexes must be removed and quantified. Therefore, these approaches require rigorous purification controls, cargo localization assays, and functional validation before they can be considered suitable for translational PELNs-based therapy [111,112].

From an engineering perspective, loading methods should be selected according to cargo properties and therapeutic goals. Incubation is suitable for small hydrophobic molecules and preserves vesicle integrity but often shows limited loading efficiency. Electroporation is useful for nucleic acids but may induce vesicle aggregation or cargo precipitation. Sonication and extrusion can improve loading of poorly permeable compounds but may alter vesicle morphology and membrane-associated proteins. Therefore, loading efficiency should not be evaluated alone; vesicle integrity, colloidal stability, retained bioactivity, release kinetics, and batch reproducibility should be reported together [94,95,96,97,98,99,100,101,102,103,104,105,106,107,108,109,110,111,112,113].

In addition to manipulating intact plant tissues, the use of plant cell suspension culture technology has brought new strategies for drug loading and vesicle engineering [114]. Similarly to mammalian cell bioreactors, plant cells can be cultured under controlled conditions, allowing for the direct secretion of pre-loaded vesicles into the extracellular media by introducing target precursor molecules or therapeutic vectors into the suspension environment. This approach bypasses the rigid cellulose boundaries of whole plant organs, significantly simplifying downstream purification workflows. Compared with physiological secretion, this top-down engineering approach can achieve a multi-fold increase in particle yield while strictly maintaining the original characteristics of the parent lipidome and phytochemicals, providing a highly feasible solution for the large-scale production of therapeutic-grade PELNs.

From a strict nanomaterial engineering perspective, the optimization of cargo encapsulation cannot be evaluated as an isolated metric. Rather, it dictates a delicate balance between loading efficiency, structural maintenance, and functional reproducibility. Passive incubation preserves the native membrane features and lipidomic integrity of PELNs but fails to achieve therapeutically relevant dosages for hydrophilic macromolecular modalities. Conversely, active platforms (electroporation, sonication, and extrusion) significantly break through the cell-membrane permeability barriers, yet the intense external physical shear or electrical fields inevitably compromise the vesicle colloidal stability, leading to membrane deformation, uncontrollable aggregation, or displacement of surface guiding proteins. Therefore, future rational design must transition from the selection of a single method toward hybrid processing strategies, such as temperature-controlled, low-voltage electroporation, or matrix-stabilized passive diffusion.

### 4.6. Surface Functionalization as Advanced Nanocarriers

To establish these engineered particles as clinical-grade, tumor-targeted nanocarriers, surface topography must be engineered to overcome biological barriers (such as the blood–brain barrier or dense extracellular matrices). Native PELNs surfaces are predominantly decorated with plant-specific carbohydrates and proteins that trigger clearance by the mononuclear phagocyte system. Recent strategies have functionalized PELN membranes with targeting ligands, including cyclic Arg-Gly-Asp (cRGD) peptides, the hemagglutinin 1 (HA1) aptamer, and folic acid [84]. These surface-functionalized nanocarriers bypass phagocytic recognition while drastically maximizing cellular uptake kinetics. This multi-stage carrier design achieves maximized local cytotoxicity while completely shielding healthy tissues from off-target toxicity.

## 5. Antitumor Applications of PELNs

PELNs have been explored for tumor therapy through two major mechanisms: as natural vesicles carrying endogenous bioactive molecules and as nanocarriers for exogenous therapeutic cargos. Their antitumor performance may involve direct cytotoxicity, apoptosis induction, immune modulation, tumor microenvironment remodeling, improved delivery of chemotherapeutic agents, and reversal of drug resistance [18,58,115,116,117,118]. However, the strength of evidence varies substantially among studies. Many reports are based on in vitro uptake and cytotoxicity assays, whereas fewer studies provide systematic biodistribution, pharmacokinetic, tumor accumulation, long-term safety, or comparative efficacy data. Therefore, antitumor applications of PELNs should be discussed not only by therapeutic mechanism, but also by evidence level, administration route, tumor model, and translational relevance [117,118].

### 5.1. Tumor-Targeting Behavior and Administration Routes

The delivery performance of PELNs is closely related to their administration route. Oral administration is attractive for edible plant-derived vesicles because it may exploit natural gastrointestinal stability, intestinal uptake, microbiota interaction, and gut–liver or gut–immune axes [87,89,96]. However, oral delivery also requires resistance to gastric acidity, digestive enzymes, bile salts, mucus barriers, and variable intestinal absorption. Intravenous administration may be more suitable for systemic tumor delivery, but it raises different challenges, including serum stability, protein corona formation, complement activation, rapid clearance by the mononuclear phagocyte system, and non-specific accumulation in the liver and spleen [117,118]. Local or transdermal administration may be useful for skin-related tumors or regional therapy, especially when vesicle stability and local retention can be maintained [74,90].

Tumor-targeting should also be interpreted carefully. Increased cellular uptake in tumor cells does not necessarily demonstrate in vivo tumor-targeting. Reliable evidence should include biodistribution analysis, tumor-to-normal tissue ratios, off-target organ accumulation, pharmacokinetics, and comparison with free drug or conventional nanocarriers. Some PELNs may show tissue preference because of their lipid composition, surface proteins, glycan structures, or plant-derived metabolites, whereas engineered PELNs may further improve targeting through membrane hybridization, ligand modification, or nucleic acid loading [76,88,117,119]. Future studies should therefore integrate route-specific design with standardized in vivo tracking and quantitative delivery assessment.

### 5.2. Intrinsic Antitumor Activity and Tumor Microenvironment Modulation

Nanocarrier-based delivery systems may improve drug solubility, stability, pharmacokinetics, and tissue distribution. However, these advantages depend on carrier composition, cargo properties, administration route, and disease model and should not be assumed for every formulation [118]. Delivery of chemotherapeutic drugs or small-molecule therapeutic agents through this system can effectively break through the limitations of traditional chemotherapy, significantly improve antitumor efficacy, and reduce systemic toxicity. Kim et al. reported that ginseng-derived exosome-like nanoparticles crossed the blood–brain barrier and exerted antitumor effects in a glioma model [11]. Bioactive components released from *Camellia sinensis*-derived TFENs could inhibit breast tumor growth [60]. Co-administration of broccoli-derived extracellular vesicles (BEVs) and 5-fluorouracil (5-FU) inhibited the proliferation and migration of HT-29 colorectal cancer cells, induced cell-cycle arrest, increased reactive oxygen species production, disrupted mitochondrial function, and promoted apoptosis [44]. These findings suggest that combining BEVs with 5-FU may improve therapeutic responses in colorectal cancer. The stilbene-derived PELNs can effectively inhibit the proliferation and migration of cancer cells, and due to their nontoxic, anticancer, and antioxidant properties, the PELNs have significant potential in the treatment of the human stromaloblastoma cell line (U87MG) [37]. PELNs have opened new avenues for tumor therapy by inhibiting the proliferation and migration of various tumor cells through multiple mechanisms. They have demonstrated therapeutic potential by inducing cell-cycle arrest and enhancing antitumor immunity. Collectively, these studies suggest that PELNs may contribute to tumor therapy by modulating tumor cell proliferation, apoptosis, immune responses, and drug sensitivity [21,44,60,64,115,116,119]. However, more systematic in vivo studies are still required to validate their biodistribution, tumor selectivity, dose–response relationships, and long-term safety.

### 5.3. Delivery of Chemotherapeutic and Phototherapeutic Agents

**Figure 5:**
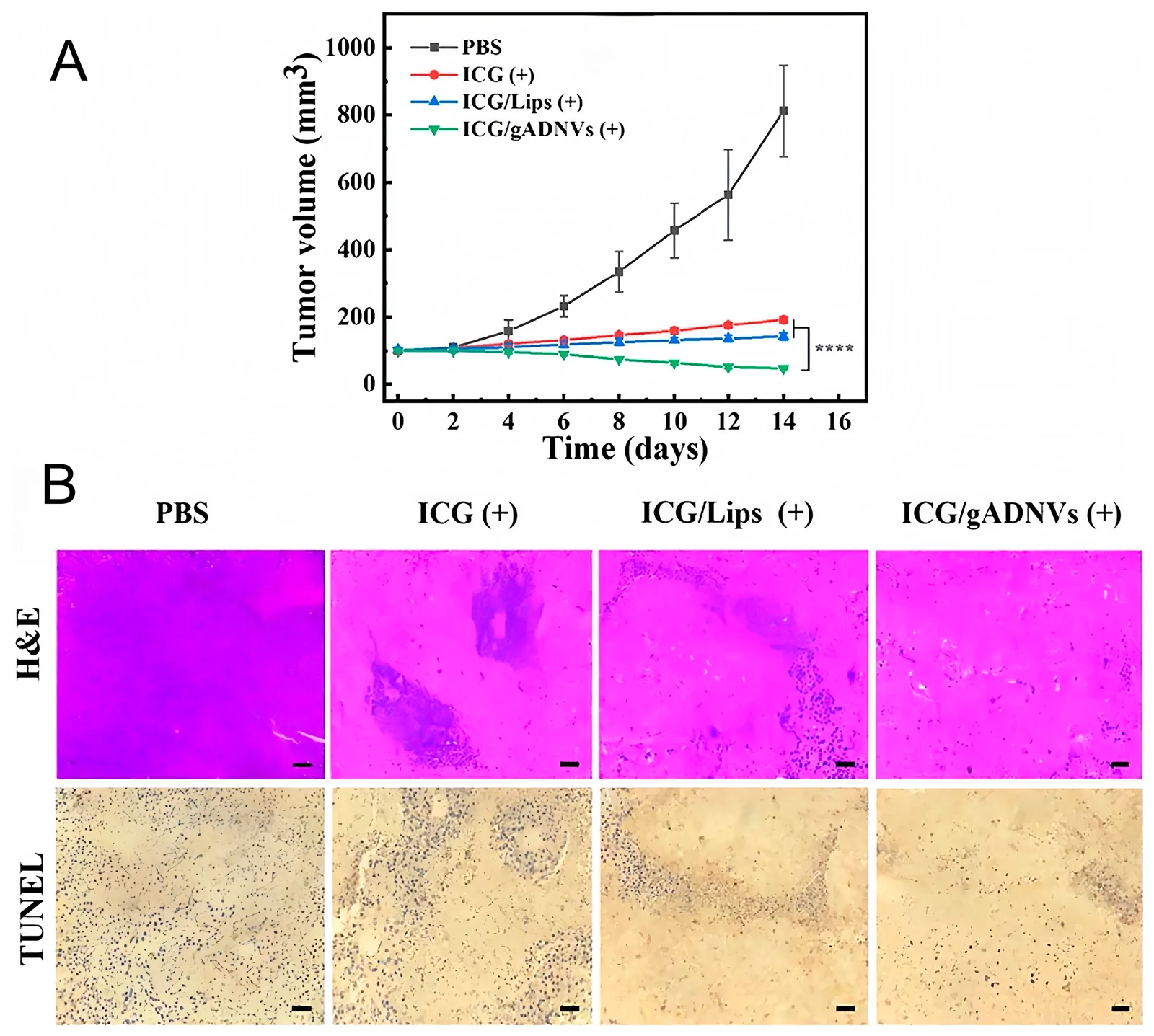
In vivo antitumor efficacy of indocyanine green-loaded aloe gel-derived nanovesicles (ICG/gADNVs) during near-infrared phototherapy. (**A**) Changes in tumor volume during treatment. Data are presented as mean ± SD (*n* = 5 mice per group). Statistical differences were analyzed using a two-tailed Student’s *t*-test. **** *p* < 0.0001 [74]. (**B**) Hematoxylin and eosin (H&E) and terminal deoxynucleotidyl transferase dUTP nick-end labeling (TUNEL) staining of tumor tissues following different treatments [74]. Scale bar = 50 μm. PBS, phosphate-buffered saline; ICG, indocyanine green; Lips, liposomes; gADNVs, aloe gel-derived nanovesicles. Reproduced from Zeng et al. [74] under the Creative Commons Attribution 4.0 International License (CC BY 4.0).

Nanodrug delivery systems have demonstrated considerable advantages in oncology by improving drug solubility, circulation behavior, tumor accumulation, controlled release, and toxicity profiles. In this context, PELNs may serve as bioinspired carriers for chemotherapeutic and phototherapeutic agents because of their lipid bilayer structure, endogenous bioactive components, and potential compatibility with oral, topical, or systemic delivery [65,74,93,120,121]. Nevertheless, their performance must be compared with free drugs and established nanocarriers under matched experimental conditions to determine whether PELNs provide genuine delivery advantages. The direct use of PELNs as drug carriers is attractive, but delivery performance should be evaluated using standardized criteria, including drug loading capacity, encapsulation efficiency, release kinetics, serum stability, cellular uptake mechanism, biodistribution, antitumor efficacy, and systemic toxicity [65,74,120,121]. Current evidence suggests that PELNs from olive, celery, aloe, basil, and Rhizoma Coptidis may improve the delivery or activity of antitumor agents, but further comparative studies are needed to clarify whether these effects arise from carrier-mediated delivery, intrinsic vesicle bioactivity, or synergistic interactions between endogenous and exogenous cargos [65,74,93,118,119]. Zeng et al. demonstrated that indocyanine green-loaded aloe gel-derived nanovesicles (ICG/gADNVs) markedly suppressed melanoma growth and induced extensive tumor-cell damage and apoptosis following near-infrared irradiation, as evidenced by reduced tumor volume and histological analyses (Figure 5) [74].

### 5.4. Reversal of Drug Resistance

Chemoresistance remains a major obstacle in cancer therapy. Tumor cells may develop multidrug resistance through enhanced drug efflux, activation of DNA repair pathways, suppression of apoptosis, metabolic adaptation, epithelial–mesenchymal transition, and microenvironment-mediated protection. Nanocarrier-based delivery may help overcome these barriers by increasing intracellular drug accumulation, protecting therapeutic cargos, enabling co-delivery of sensitizing agents, and modulating resistance-related signaling pathways [44,50,76,122].

Delivery of chemotherapeutic drugs or small-molecule inhibitors based on the nano-delivery system NDS can significantly improve drug bioavailability, extend circulating half-life, and reverse multidrug resistance, providing an innovative solution for oncology treatment. A variety of PELNs show great potential in this field. Cao et al. found that broccoli-derived extracellular vesicles (BEVs) reversed 5-FU resistance in HT-29 cells by inhibiting the aberrant activation of the PI3K/Akt/mTOR signaling pathway, a finding that provides a new idea for overcoming chemotherapy resistance in rectal cancer [44]. The combination of siRNA targeting integrins and quercetin can enhance the efficacy of quercetin in reversing cancer drug resistance, opening up new avenues for cancer treatment. This combined therapeutic strategy makes full use of the synergistic effects of different drugs to improve the therapeutic efficacy and reduce the side effects of drugs [122]. Bitter melon-derived EVs reduced the resistance of oral squamous cell carcinoma (OSCC) to 5-FU and exerted synergistic therapeutic effects by significantly down-regulating the expression of NLRP3. Yang et al. [50] proposed that the RNA of BMEVs at least partially mediated anti-inflammatory bioactivities. The activation of NLRP3 contributes to the resistance of OSCC cells to 5-FU. BMEVs were also found to enhance the therapeutic effects of 5-FU against OSCC both in vitro and in vivo. The kiwifruit-derived EV (KEV), which has a high safe dose and can be further loaded with siSTAT3, confers EGFR targeting ability and fluorescence. Its systemic administration significantly inhibits tumor xenografts through STAT3-mediated apoptosis, making it a next-generation gene delivery platform for multidrug-resistant therapy in non-small cell lung cancer (NSCLC). This safe and powerful KEV has become a next-generation gene delivery platform for NSCLC treatment after multi-drug resistance, bringing a new change to lung cancer treatment [76].

These studies indicate that PELNs may contribute to drug resistance reversal by improving drug delivery, modulating inflammatory signaling, altering apoptosis-related pathways, or enabling nucleic acid-based intervention [44,50,76,122]. However, resistance reversal should be validated using well-defined resistant cell lines, matched parental controls, in vivo resistant tumor models, and mechanistic rescue experiments. Without these controls, it remains difficult to distinguish true resistance reversal from general cytotoxic enhancement.

### 5.5. Synergistic Enhancement of Antitumor Efficacy

PELNs may be useful for combination therapy because they can carry endogenous bioactive components and exogenous therapeutic cargos simultaneously. Potential synergistic strategies include combination with chemotherapy, photodynamic therapy, immunotherapy, hypoxia modulation, and microbiota regulation [91,123,124]. For example, plant-derived photoactive compounds or vesicle-associated metabolites may support phototherapeutic strategies, while loaded chemotherapeutics may benefit from vesicle-mediated uptake or improved stability. However, examples based on non-PELNs synthetic nanoparticles should be discussed only as conceptual references and should not be presented as direct evidence for PELNs efficacy [123,124].

Future combination studies should distinguish additive effects from true synergy using appropriate combination index analysis, dose-matched controls, and mechanistic validation. In addition, the contribution of endogenous PELNs cargo, exogenous therapeutic cargo, and carrier structure should be separately evaluated to clarify the basis of enhanced antitumor activity [90,123,124].

## 6. Conclusions

PELNs represent a promising class of bioinspired nanocarriers with structural stability, endogenous bioactive cargos, and potential compatibility with tumor drug delivery. Compared with some synthetic or mammalian-derived nanocarriers, PELNs offer potential advantages in source availability, biocompatibility, and intrinsic biological activity. However, their development as reproducible therapeutic platforms requires a clearer understanding of how plant source, isolation workflow, vesicle composition, cargo-loading method, and administration route collectively determine delivery performance [125].

From a source, processing, structure, and performance perspective, several challenges remain unresolved. First, preparation and purification methods are not standardized, and different workflows may produce vesicle preparations with different purity, size distribution, cargo profiles, and functional activities. Second, reliable plant-specific EV markers and universal quality-control standards are still lacking, making it difficult to compare PELNs across studies [126]. Third, cargo-loading efficiency remains variable, and loading methods may alter vesicle morphology, membrane integrity, and bioactivity. Fourth, storage and transport conditions require optimization because temperature, pH, freezing, lyophilization, and excipients may affect vesicle stability and function [127]. Unlike synthetic lipid nanoparticles (LNPs) composed of defined excipients, PELNs exhibit a complex dual-modality interface consisting of diverse endogenous phospholipids, glycolipids, phytochemicals, and small RNAs. This structural complexity seriously complicates mechanistic interpretation and clinical trial batch-consistency validation. From a safety perspective, although plants lack mammalian zoonotic pathogens, the heterologous proteins and unique plant-specific lipids could trigger distinct host immune recognition pathways. Particularly via intravenous deployment, these components pose potential risks of activating the complement cascade or inducing accelerated blood clearance (ABC) upon repeated administration. Furthermore, the cross-kingdom gene regulation potential of endogenous plant miRNAs remains controversial due to missing standardized RNase protection metrics and target-validation consensus, leaving their long-term clinical safety and off-target biological risks unresolved.

Clinical translation of PELNs-based formulations is further constrained by batch-to-batch variability, incomplete safety assessment, uncertain pharmacokinetics, limited biodistribution data, and ambiguous regulatory classification [128,129]. Although early clinical studies involving grape-, ginger-, or aloe-derived vesicle-like nanoparticles have been registered, the field remains at an early stage and lacks sufficient evidence for human efficacy [130]. Future studies should, therefore, prioritize standardized reporting of plant source, isolation method, particle yield, purity, protein-to-particle ratio, cargo composition, sterility, storage stability, administration route, biodistribution, and toxicological profile.

In the next stage, PELNs research should move beyond descriptive characterization and toward rational formulation design. GMP-compatible isolation workflows, route-specific delivery strategies, validated tumor-targeting assays, quantitative loading and release studies, and long-term safety evaluation will be essential. If these issues are addressed, PELNs may become valuable candidates for developing natural nanocarrier-based tumor therapies, either as intrinsic bioactive vesicles or as engineered carriers for chemotherapeutic agents, nucleic acids, immunomodulators, and combination therapies [131,132].

## Figures and Tables

**Table 1:** Extraction methods, sources, and characterization of different PELNs.

Isolation Method	PELNs Source/Type	Size	Purity/Concentration	Zeta Potential	Refs.
Ultracentrifugation	Garlic PELNs	50–150 nm	/	/	[24]
	Ginseng-derived nanoparticles (GDNPs)	200.4–344.8 nm	/	−25.4 mV	[22,59]
	Asparagus cochinchinensis-derived nanovesicles (ACNVs/PEG-ACNVs)	108 ± 17 nm	/	−10 mV	[25]
	Cabbage-derived nanovesicles (Cabex/Rabex)	134.2 nm	0.432 × 10^9^ particles/μg protein	Cabex: −14.8 mV; Rabex: −15.2 mV	[35]
	Camellia sinensis-derived TFENs	Mean hydrodynamic size: 131 nm	Proteins > 15 kDa	/	[60]
	*Portulaca oleracea* L.-derived PELNs	160 nm	/	−31.4 mV	[61]
Polymer precipitation	*Solanum nigrum* L.-derived PELNs	Average size: 107 nm; modal size: 139 nm	8.1 × 10^9^ particles/mL	−0.6 mV	[27]
	Green onion-derived GDENs	167.4 nm	/	−16.06 mV	[28]
	Papaya-derived PELNs	160.95 ± 9.45 nm	/	−9.4 ± 0.2 mV	[31]
	Yam bean root-derived PELNs	236.83 ± 9.27 nm; spherical morphology	/	−26.5 mV	[33]
	Cabbage-derived nanovesicles (Cabex/Rabex)	148.2 nm	0.242 × 10^9^ particles/μg protein	Cabex: −14.8 mV; Rabex: −15.2 mV	[35]
	*Atractylodes macrocephala*-derived AMEVLPs	90.61 ± 19.66 nm; round or cup-shaped morphology	6.27 × 10^11^ particles/mL	/	[62]
Size-exclusion chromatography	Cabbage-derived nanovesicles (Cabex/Rabex)	98.8 nm	10 × 10^9^ particles/μg protein	Cabex: −14.8 mV; Rabex: −15.2 mV	[35]
	Carrot-derived Carex	150 nm	/	−10.2 mV	[36]
	*Viburnum opulus*-derived VDNVs	45.36 nm by DLS	/	−2.87 mV	[37]
	*Hypericum perforatum*-derived HPELNs	67 ± 3.13 nm	/	−16.4 ± 0.28 mV	[63]
Extraction kit	Ginger-derived GELNs	156 ± 36 nm	/	−26.6 ± 5 mV	[43]
	Broccoli-derived BEVs	133.3 ± 66.4 nm	Protein concentration: 2056.05 ± 35.27 μg/mL	15.60–9.86 mV	[44]
Ultrafiltration	*Viburnum opulus*-derived VDNVs	45.36 nm by DLS	/	−2.87 mV	[37]
	Corn-derived cNPs	80 nm	Approximately 35.6 ± 1.9 × 10^11^ particles/mL	−17 mV	[46]
	Perilla leaf-derived PELNs	100 nm; spherical morphology	/	−12.3 mV	[48]
	Rice bran-derived rbNPs	139.0 ± 1.3 nm	Approximately 4 × 10^13^ PELNs/100 g rice bran	−17.2 ± 2.2 mV	[64]
	Rhizoma Coptidis-derived natural nanoparticles	138.6 ± 8.2 nm	/	−20.8 ± 0.6 mV	[65]

**Table 2:** Composition and antitumor applications of different PELNs.

Name	Composition	Therapeutic Application	Refs.
Artemisia annua-derived ADNVs	Mitochondrial DNA (mtDNA)	Remodel the tumor microenvironment and reprogram tumor-associated macrophages, thereby inhibiting tumor growth and enhancing antitumor immunity.	[21]
Garlic-derived PELNs	Heat shock protein 70 (HSP70), CD9, and CD63-associated proteins	Exert anticancer effects by inducing caspase-mediated apoptosis.	[24]
Edible asparagus-derived ACNVs	20–250 kDa proteins, multiple lipids, and small RNAs	Accumulate at tumor sites, inhibit tumor growth, and enhance apoptosis.	[25]
Coffee-derived PELNs	Mainly miRNAs	Inhibit the proliferation of hepatocellular carcinoma cells.	[26]
*Solanum nigrum* L. berry-derived PELNs	2-Amino-5-methylbenzoic acid, eicosane, heneicosane, 5-(2-methylpropyl)nonane, palmitaldehyde, tricosane, and pentacosane	Exert anti-inflammatory effects by inhibiting LPS-induced inflammatory responses.	[27]
Bitter melon-derived BMEVs	MAP30 protein	Induce ROS-mediated apoptosis in cancer cells.	[50]
Camellia sinensis-derived TFENs	Proteins > 15 kDa; lipids including PC, TG, and PE	Inhibit breast cancer growth and metastasis and regulate intestinal flora.	[60]
Rice bran-derived rbPELNs	Lipids including LPC, PCs, PEs, PSs, and sphingomyelins (SMs)	Inhibit cancer cell proliferation and peritoneal spread.	[64]
Tea-derived TLNTs	Proteins involved in cellular metabolism	Increase ROS levels in breast cancer cells and induce mitochondrial damage and apoptosis.	[69]
Tangerine-derived nanovesicles (TNVs)	Lipids, citrullinated compounds, and flavonoid derivatives	Deliver DDHD1-siRNA and mediate targeted inhibition of cancer-related gene expression.	[72]
Prevent oxidative stress in human mesenchymal stromal cells	miRNAs	Regulate plant development and immunity.	[81]
Cucumber-derived CDNVs	/	Increase ROS levels and activate the caspase pathway to induce apoptosis and anticancer effects.	[88]
Mulberry-derived MLPELNs	Lipids, proteins, and flavonoids	Increase ROS levels in hepatocellular carcinoma cells, inhibit tumor proliferation and metastasis, and regulate intestinal microbiota balance.	[89]
Grapefruit-derived GEVs	HSP70	Stimulate CD8+-dependent antitumor immunity and modulate innate immune responses against colorectal cancer cells.	[91]
Bitter melon-derived MC-ELNs	miRNAs, with miR5266 showing the highest expression	Reduce infarct size and improve ischemia–reperfusion-induced neurological injury.	[92]
Basil leaf-derived EVs	/	Exert potent anticancer effects in pancreatic cancer cells.	[93]

**Table 3:** Comprehensive comparison of engineered PELNs platforms, loading parameters, and therapeutic outcomes.

Botanical Source	Core Engineering Strategy	Encapsulated Cargo	Loading Efficiency (%)	Targeting Modality/Surface Modifiers	Intended Oncological Application and Therapeutic Efficacy	Refs.
Grapefruit (GEVs)	Overnight incubation followed by 15 min sonication and ultrafiltration	Recombinant HSP70	Approximately 0.5 μg HSP70 per 10^11^ GEVs	/	CD8^+^-associated antitumor immunity in colon cancer	[91]
Ginger (GDNVs)	Isolation and purification by sucrose-gradient ultracentrifugation	Endogenous 6-gingerol and 6-shogaol	Not reported	Oral administration and colon targeting	Prevention and treatment of colitis and colitis-associated cancer	[86]
Lemon (LEDNs)	Electrophoretic technique with 300 kDa cut-off dialysis bag	Intrinsic components	/	Gastrointestinal retention	S-phase arrest and ROS-mediated apoptosis in gastric cancer	[49]
Broccoli (BSDExo)	Co-administration of BEVs with free 5-fluorouracil	No encapsulated cargo; 5-FU was administered separately	/	/	Restoration of 5-FU sensitivity via PI3K/Akt/mTOR inhibition.	[44]
Kiwi fruit 00(KEVs)	siSTAT3	siSTAT3	57.50%	EGFR-targeted aptamer functionalization	Suppressed subcutaneous PC9-GR4-AZD1 tumor xenografts in nude mice by STAT3 mediated apoptosis	[76]

## Data Availability

No new data were created or analyzed in this study. Data sharing is not applicable to this article.

## Abbreviations

PELNs	Plant-derived exosome-like nanoparticles
EVs	Extracellular vesicles
UC	Ultracentrifugation
DG-UC	Density-gradient ultracentrifugation
SEC	Size-exclusion chromatography
UF	Ultrafiltration
TFF	Tangential flow filtration
PEG	Polyethylene glycol
TEM	Transmission electron microscopy
DLS	Dynamic light scattering
miRNA	MicroRNA
DOX	Doxorubicin
DTX	Docetaxel
TME	Tumor microenvironment
MDR	Multidrug resistance
PDT	Photodynamic therapy
ICG	Indocyanine green
